# A Hand-Book of Therapeutics

**Published:** 1872-03

**Authors:** 


*A Hand-Book of Therapeutics.* By SIDNEY RINGER, M. D., etc. New York, Wm. Wood & Co., 1871.

The author in the new edition of his Hand-Book of Therapeutics, has made a very thorough revision of the work, and has added much to it which is of value. He has followed the plan of Buchheim, and has produced a work of much value to the profession. That this is the case seems to be proved by the short time which has transpired, (about a year ago, we believe,) since the first edition was published. The present one contains much additional matter, and having undergone revision, and the corrections, which experience would indicate, comes to us in the form of a very useful work on therapeutics.

